# CRISPR/Cas *StNRL1* gene knockout increases resistance to late blight and susceptibility to early blight in potato

**DOI:** 10.3389/fpls.2023.1278127

**Published:** 2024-01-18

**Authors:** Moshen Norouzi, Farhad Nazarain-Firouzabadi, Ahmad Ismaili, Rahim Ahmadvand, Helen Poormazaheri

**Affiliations:** ^1^ Production Engineering and Plant Genetics Department, Faculty of Agriculture, Lorestan University, Khorramabad, Iran; ^2^ Associate Professor, Seed and Plant Improvement Institute, Agricultural Research, Education and Extension Organization, Karaj, Iran; ^3^ Department of Biology, Shahr-e-Qods Branch, Islamic Azad University, Tehran, Iran

**Keywords:** *Alternaria alternata*, CRISPR/Cas9, effector Pi02860, NPH3/RPT2-LIKE1 protein, *Phytophthora infestans*, susceptible gene

## Abstract

With the development of genome editing technologies, editing susceptible genes is a promising method to modify plants for resistance to stress. *NPH3/RPT2-LIKE1* protein (*NRL1*) interacts with effector Pi02860 of *Phytophthora infestans* and creates a protein complex, promoting the proteasome-mediated degradation of the guanine nucleotide exchange factor SWAP70. SWAP70, as a positive regulator, enhances cell death triggered by the perception of the *P. infestans* pathogen-associated molecular pattern (PAMP) INF1. Using a clustered regularly interspaced short palindrome repeats (CRISPR)/CRISPR-associated protein 9 (Cas9) system, a construct was made to introduce four guide RNAs into the potato cultivar Agria. A total of 60 putative transgenic lines were regenerated, in which 10 transgenic lines with deletions were selected and analyzed. A mutant line with a four-allelic knockdown of *StNRL1* gene was obtained, showing an ~90% reduction in *StNRL1* expression level, resulting in enhanced resistance to *P. infestans*. Surprisingly, mutant lines were susceptible to *Alternaria alternata*, suggesting that *StNRL1* may play a role as a resistance gene; hence, silencing *StNRL1* enhances resistance to *P. infestans*.

## Introduction

Potato (*Solanum tuberosum*) is the third most important and strategic food crop in terms of global food production and security, with more than 370 million tonnes produced in 2019 (FAO, 2020). It is a major and irreplaceable portion of the human diet worldwide (Eriksson et al., 2016; Kieu et al., 2021). Potato tubers are rich in carbohydrates and vital elements, including minerals, vitamins, and fibers (Yang et al., 2021).

Potato farms suffer from devastating plant diseases caused by fungi, oomycetes, bacteria, and viruses. Potato late blight, caused by *Phytophthora infestans*, is one of the most infamous potato diseases worldwide and in Iran, especially in Golestan Province (Aghajani, 2012). Late blight continues to be the main disease of potato with an annual loss of approximately €6.1 billion, posing a great danger to food security, especially in poor and developing countries (Adolf et al., 2020). Given favorable environmental conditions like moderate temperatures and moderate to high humidity, an asexual cycle of sporangial proliferation is completed within only 5 days, leading to the destruction of an unprotected potato field within 10 days after infection with *P. infestans* (Fry, 2008).

Application of chemical fungicides is the most common strategy to control late blight, hence making this disease a billion-dollar business for fungicide manufacturers. Despite the effectiveness of fungicides, emerging new fungicide-resistant isolates challenge plant breeders to look for genetic approaches to limit late blight severe yield loss (Rosenzweig et al., 2008; Fisher et al., 2012). Today, perhaps the most elegant strategy to control *P. infestans* is the use of potato resistance cultivars. However, breeding resistant cultivars continues to be a major and challenging task for traditional plant breeders as it is time consuming and difficult in potatoes with vegetative nature of propagation (Richael, 2021).

The plant resistance genes (R-genes) confer resistance to devastating pathogens by gene-for-gene strategy. Stacking several late blight resistance genes from different potato wild species led to an elevated late blight resistance in potato cultivars (Zhu et al., 2012; Ghislain et al., 2019). Despite introducing 11 major R-genes from *Solanum demissum* to potato cultivars, resistance was quickly overcome by the evolution of *P. infestans* isolates in nature (Fry, 2008).

Susceptible genes (S-genes) facilitate infection and support compatibility and hence provide pathogens with unique advantages to colonize host plants (van Schie and Takken, 2014). The resistance conferred by R-genes and the loss of function of S-genes are generally dominant and recessive, respectively. Based on molecular and biological functions, S-genes have been divided into three major groups (van Schie and Takken, 2014; Kieu et al., 2021). Pathogens employ the first group of S-genes to establish recognition and consequently penetration into host cells. One typical susceptibility gene is the powdery mildew resistance locus O (*MLO*) gene. *MLO* encodes a membrane-anchored protein that is required for full susceptibility to powdery mildew infection (van Schie and Takken, 2014). The role of *MLO* in powdery mildew susceptibility has been confirmed in *Arabidopsis*, pepper, cucumbers, tomato, wheat, and melons (van Schie and Takken, 2014). The second group of S-genes includes genes mostly involved in pathogen sustenance by providing pathogens with nutrients and facilitating amino acid uptake, which is vital for pathogen metabolite biosynthesis pathways and sugar transport (van Schie and Takken, 2014). *SWEET11* and *SWEET13* genes in rice are sugar transporters encoding *SWEET* proteins, which transport sugar to apoplastic space for *Xanthomonas oryzae* growth and development (Streubel et al., 2013). The third group of S-genes encode negative regulators of plant immune signaling. For instance, the cullin-based E3 ligase BTB domain containing NPH3/RPT2-LIKE1 protein (*NRL1*), which forms a complex with the *P. infestans* Pi02860 effector, promotes the proteasome-mediated degradation of the guanine nucleotide exchange factor SWAP70 (Yang et al., 2016; He et al., 2018). SWAP70 regulates immune responses through cell death triggered by the perception of the *P. infestans* pathogen-associated molecular pattern (PAMP) INF1, thus leading to lower leaf colonization (Yang et al., 2016; He et al., 2018). Therefore, knocking out the *NRL1* gene as a key negative regulator can suppress degradation of SWAP70 protein and increase resistance to late blight disease.

Although some S-genes in potatoes (*StDND1* and *StDMR6*) have been knocked down by RNA interference (RNAi) technology, resulting in enhanced late blight resistance, it is known that in RNAi transgenic lines, undesirable phenotypes appear and silencing of a transgene itself may lead to aberrant consequences (Sun et al., 2016).

Clustered regularly interspaced short palindrome repeats (CRISPR)/CRISPR-associated protein 9 (Cas9) is a powerful genome editing tool in plants to improve crop plants with high potential to combat devastating pathogens (Chen et al., 2019). CRISPR/Cas has frequently been used to knock out several S-genes in potatoes, including *StDND1*, *StCHL1*, and *StDMR6-1*, increasing resistance to late blight (Kieu et al., 2021). In this study, NLR1 gene as an important S-gene in potato was knocked out by CRISPR/Cas9.

## Materials and methods

### Design of gRNAs and vector constructs

The full-length sequence of the potato *StNLR1* gene (accession no. Sotub02g031050.1.1) was retrieved from a potato genome database. Four single guide RNAs (sgRNAs) (sgRNA1: 5′-TGCCTTGCTGAGATGCGCGG-3′; sgRNA2: 5′-AATATCCCTGATATACCTGG-3′; sgRNA3: 5′-GATGTAACTGTAAATGCAGG-3′; and sgRNA4: 5′-AGAGGTCCTCTTCATAGCAG-3′) were designed according to Liang et al. (2016). The first list of sgRNAs was predicted using the CC-Top CRISPR/Cas9 Target Prediction Tool (Stemmer et al., 2015). The G+C content and folding of sgRNAs were also evaluated using ENDMEM (http://www.endmemo.com/bio/gc) and the MFOLD web server (Zuker, 2003), respectively. Cas-OFFinder was used to investigate the potential off-target effects of the four sgRNAs. (Bae et al., 2014). PCR was performed to analyze possible off-target mutations in genomic sequence using flanking and specific primers amplifying regions from predicted off-target genes.

**Figure 1:**
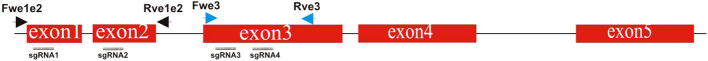
The schematic structure of NPH3-RPT2-Like gene (*StNRL1*) showing the position of target sites of the four designed sgRNAs and the flanking primers used for screening and Sanger sequencing.

sgRNA1 and sgRNA2 were designed to target the first and the second exons, respectively, and sgRNA3 and sgRNA4 were also directed to the third exon of *StNLR1* gene (**Figure 1**). A set of Addgene plasmids was used (ordered by the Wageningen University Plant Breeding Lab, the Netherlands) to assemble the CRISPR constructs by Golden Gate cloning technology according to the described protocols (Weber et al., 2011; Marillonnet and Werner, 2020). Briefly, the pICH86966 plasmid was used as a template for the amplification of the four sgRNAs. Each of the four sgRNAs driven with the U6-26 promotor of the pICSL01009 plasmid was assembled in the level 1 plasmids (pICH477751, pICH47761, pICH47772, and pICH47781) using the *BsaI* restriction enzyme. Next, the level 1 plasmids were combined with pICH47732 (NPTII gene donor), pICH47742 (CAS9 gene donor), and pICH41822 (as linkers) to construct the level 2 plasmid, pAGM4723, using the *BpiI* restriction enzyme (**Supplementary Figure 1**). The final level 2 plasmid was introduced to *Agrobacterium tumefaciens* strain AGL1 for transformation.

### Potato transformation and tissue culture

Tetraploid *S. tuberosum* cv. Agria (susceptible to late blight disease) was obtained from the Crop Research Department (Seed and Plant Breeding Research Institute, Karaj, Iran). The Agria seedlings were maintained on Murashige and Skoog (MS) basal medium including vitamins (Duchefa, M0222.0050) with 30 g/L sucrose and 7.5 g/L agar. Plants were incubated at 24 ± 2°C under a 16/8 h (day/night) photoperiod. The internode explants (2–5 mm) of 4-week-old *S. tuberosum* cv. Agria were transformed according to a protocol described by Banerjee et al. (2006). In short, explants were incubated in a liquid MS medium for 48 h in the dark. Next, the explants were transferred to an *A. tumefaciens* (OD600 = 0.9) harboring pAGM4723 construct suspension for transformation for 5–10 min. Next, explants were transferred to MS base medium containing 2.5 mg/L zeatin riboside (cat.: Z0375), 0.01 mg/L 1-naphthaleneacetic acid (NAA) (cat.: N0640), and 0.03 mg/L Gibberellin A3 (GA3) hormones (cat.: G7645) and 200 mg/L kanamycin (cat.: K1377), cefotaxime (cat.: C7039), and vancomycin (cat.: SBR00001) each. After proper callus formation in weeks 3 to 4, explants were transferred to a culture medium containing 2.5 mg/L zeatin riboside for 1 week. Explants were subcultured onto fresh media every 10–14 days to maintain selection pressure. Shoots that emerged after 4–5 weeks were cut and transformed into fresh MS medium. An MS medium with no growth hormone was used to root the plantlets. Rooted shoots were selected and transferred onto the sterile soil, and finally, after a 2-week hardening period, they were transferred to a greenhouse for further analyses.

### PCR screening of mutant lines and sequencing

The screening of T1 potato mutant lines with possible alteration in the *StNLR1* gene was performed by the PCR analyses, using specific primers (F1: 5′-ACTGAGATGTGACTAAGGT-3′ and R1: 5′-GATAACAATGGATTCCTCCA-3′, F2: 5′-TCTGCTCTACCTGATGTGG-3′ and R2: 5′-TCACGCTCACACCTTAAC-3′). The PCR products were both electrophoretically analyzed and Sanger sequenced.

### Extraction of RNA and quantitative PCR analyses

Total RNA was extracted using TRIzol reagent (Invitrogen, Carlsbad, California, United States; cat.: 15596026) according to the manufacturer’s instruction. The quality and quantity of total RNA were assessed using agarose gel electrophoresis and NanoDrop spectrophotometer analyses, respectively (**Supplementary Figure 2**). Complementary DNA (cDNA) synthesis was performed using the SinaClon cDNA synthesis protocol (SinaClon, Tehran, Iran; cat.: RT5201). Quantitative PCR (qPCR) was performed using F: 5′-TTAGAACTGGATGTGCCTTC-3′ and R: 5′-AATAGCCTCTGACCGTAATC-3′ primers to analyze the expression level of the *StNLR1* gene. Prior to real-time reverse transcription PCR (RT-PCR) analyses, the efficiency of specific primers was assessed. qPCR analyses were done in three replicates using SYBR Green. A 10-μl total reaction volume includes 100 nM of each primer, 2 μl of diluted cDNA template, and 2.5 μl Master Mix reaction buffer. The thermal cycling conditions were as follows: initial denaturation step at 95°C for 3 min followed by 40 cycles (denaturation at 95°C for 10 s, annealing at 60°C for 10 s, and extension at 72°C for 20 s). The elongation factor gene (*elf*1-α) was used as an internal control (Nicot et al., 2005). In order to analyze the qPCR data, the mean of threshold cycle (C_t_) values was normalized between treated and control samples using the 2^−ΔΔCt^ formula (Livak and Schmittgen, 2001). A Student’s t-test analysis (P ≤ 0.01) was used to compare the means of the expression level of different transgenic lines with that of the control plant.

### Detached leaf assay with *P. infestans*

Isolates of *P. infestans* were obtained from potato fields (Golestan Province, Iran). *P. infestans* was grown on rye B agar medium at 16°C in the dark for 10 days. Sporulating mycelia were flooded with 20 ml ice-cold sterile water, adjusted to 50 sporangia per microliter, and the suspension was incubated for 2 h to 3 h at 7°C and then for 0.5 h at room temperature (Sobkowiak and Śliwka, 2017). The detached leaves were inoculated with 10 μl of zoospore suspension on the abaxial side of the leaves in Petri dishes. A total of 12 spots on each leaf were inoculated with the zoospore suspension. The leaves were stored for 5 days in a closed box at 18°C. The leaves were then scored on a scale of 1 to 10 for each leaf, where 10 was designated for asymptomatic leaves; 9 for lesions not larger than the inoculation drops; 8 for lesions up to 0.5 cm in diameter; 7 for diffuse lesions up to 1 cm in diameter with no spores and water soaking; 5 for lesions larger than 1 cm, with no spores; 4 for large lesions impregnated with water with spores visible only through binocular microscope; 2 for large lesions macroscopically visible on one side of the leaves; and, finally, 1 for large lesions with spores that can be seen macroscopically on both sides of the leaves (Monino-Lopez et al., 2021). The average disease index scores were compared by one-way ANOVA followed by Fisher’s least significant difference (LSD; P ≤ 0.05).

### Detached leaf assay with *Alternaria alternata*

Conidial spores of *Alternaria alternata* were obtained from naturally infected potato plants. The infected leaves were put on water, and after 2 days, cultures were grown on potato dextrose agar (PDA) medium (2% agar) under 24-h lights at room temperature. For the detached leaf assays, plants were grown in pots in a glasshouse (for 7 weeks). For the fungus resistance assays, three terminal leaflets from the fully developed leaves at the middle part of the stem were detached from each of three plants of selected T1 potato lines. The leaflets were placed on wet filter papers in trays adaxial side down, and the leaf surfaces were wounded by a glass rod and inoculated by a square of media (~3 mm^−2^) of 10-day-old *A. alternata* culture. After 10 days, the lesion areas were analyzed using a 0 to 11 score chart (Abubakkar et al., 2010) with slight modifications: 0 = no disease; 1 = less than 1% leaf tissue diseased; 3 = small brown to dark brown necrotic lesions, 1% to 10% diseased; 5 = necrotic lesions, 11% to 25% diseased; 7 = necrosis lesions, 26% to 50% diseased; 9 = necrosis lesions, 51% to 70% diseased; and 11 = necrosis lesions, >71% diseased. The average disease index scores were compared by one-way ANOVA followed by Fisher’s LSD (P ≤ 0.05).

## Results and discussion

In order to produce mutants in potato *NRL1* gene (Sotub02g031050.1.1) (XM_006339754.2), a single CRISPR/Cas9 construct containing four sgRNAs was made and introduced to potato cultivar Agria via *Agrobacterium*-mediated transformation (Kusano et al., 2018). sgRNA1 and sgRNA2 were designed to disrupt the BTB/POZ domain (exone 1 and 2) in the N-terminal region, and sgRNA3 and sgRNA4 were targeted to the NPH3 domain of *StNRL* gene (**Figure 1**).

**Figure 2:**
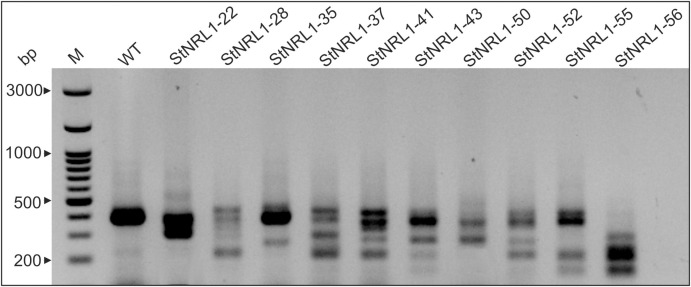
Characterization of *StNRL1* knockout CRISPR mutant lines in potato. PCR screening of mutant lines by amplification of the *StNRL1* knockout allele. M, 100-bp size marker; WT, wild-type control plant.

In total, 60 putative mutants were generated and screened by PCR analyses. Knockout lines were designated as *StNRL1*-xx, in which xx denotes the line number. For screening large INDLs (deletions/insertions), the PCR product length was compared with that of the wild-type (WT) controls. Out of 60 regenerated plants (T1), 10 T1 lines for site 1 and three T1 lines for site 2 displayed a PCR product length smaller than that of PCR product length of control plants, respectively, suggesting possible fragment deletions (**Figure 2**). Only *StNRL1*-56 with possible tetra-allelic deletion was found, and then *StNRL1*-56 and *StNRL1-22* with two bands were selected as possible biallelic mutants for further analyses.

### Characterization of CRISPR/Cas9-mediated *StNRL1* mutants

**Figure 3:**
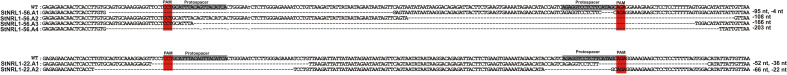
Sequence analyses of the target sites in one homozygous *NRL1* (tetra-allelic knockout line) and one heterozygous line (biallelic knockout line). The WT sequence is shown at the top of the mutant alleles. The protospacer adjacent motif (PAM) and protospacer sequences are shown and highlighted in gray and red, respectively. Black short-dashed lines indicate deletions. The sequence gap length is also mentioned on the right side of the corresponding sequences.

*StNRL1*-56 and *StNRL1*-22 mutants with the *StNRL1* locus showing PCR product length differences were selected and Sanger sequenced (**Figure 3**). Compared to the WT allele, *StNRL1*-56 showed a relatively large deletions at both site 1 and site 2. In mutant line *StNRL1*-56, large deletions with almost 100 nucleotides occurred for all four alleles. Plus, one allele exhibited a further four-nucleotide deletion. Similar to the *StNRL1*-56 mutant line, deletions were also observed for two alleles of the *StNRL1*-22 mutant line (**Figure 3**). Since PCR screening of mutants was not able to detect short INDLs as well as point mutations, the number of potato mutants lines detected was relatively lower as compared to that of other screening methods (Johansen et al., 2019). Similarly, Kieu et al. (2021) concluded that using constructs expressing two gRNAs targeting a larger segment of target genes is in favor of PCR screening as a low-cost and fast method to select mutants. Although two sgRNAs were designed to target different *StNRL1* domains at both termini, no mutant lines were detected showing INDLs at both sites.

In order to reduce the chance of undesired mutations, designing the sgRNAs with minimal chance of off-targets is a key determinant of CRISPR technology success. The Cas-OFFinder program was used to predict the off-target sequences with up to three mismatches (Bae et al., 2014). A putative off-target with up to three mismatches was predicted for all four gRNAs targeting a gene encoding a root phototropism protein (PGSC0003DMG400024968). An NB-ARC domain-containing protein (PGSC0003DMG400041909) and a BZIP domain class transcription factor (PGSC0003DMG400014877) were also predicted as off-targets for sgRNA2 and sgRNA3, respectively. PCR size analyses as well as sequencing data did not show any mutation in the putative off-target genes (data not shown).

**Figure 4:**
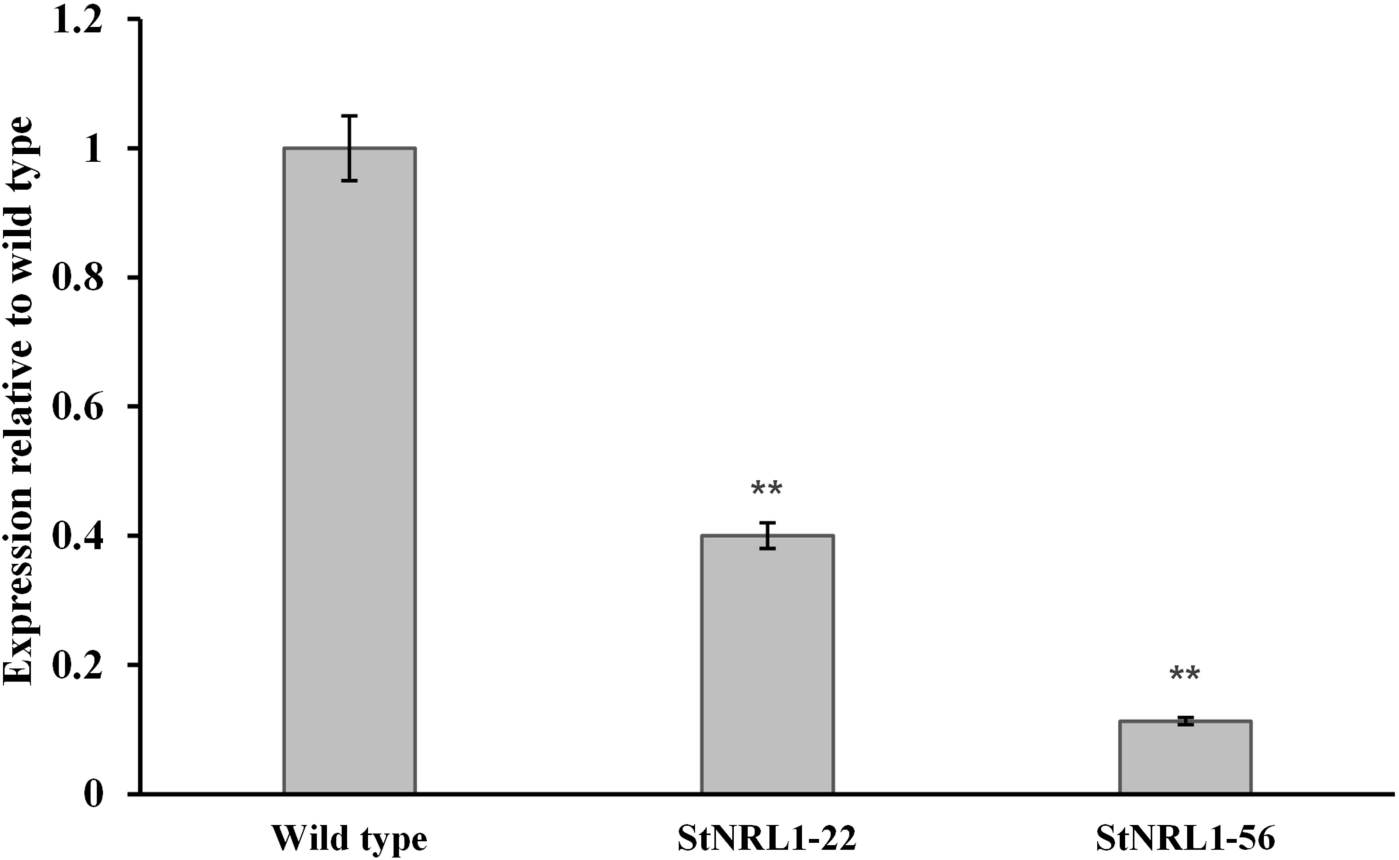
*StNRL*1 gene expression level in the tetra-allelic mutant plants (*StNRL1*-56) and the *StNRL1*-22 heterozygous line in comparison to the wild-type plant (Agria). For the relative expression of *StNRL1* gene by qRT-PCR, the ΔCt values of transgenic plants were compared to the ΔCt values of control potato plants using Student’s t-test. **: significant at 1% probability level.

In order to see whether the knocking-out procedure results in reduction of *StNRL1* gene expression, *StNRL1*-56 and *StNRL1*-22 mutants were subjected to real-time RT-PCR analyses. Student’s t-test analyses (P ≤ 0.01) revealed a significant expression difference (s) among transgenic lines with that of the control plants (**Figure 4**). The results showed that *StNRL1* expression decreased by 90% and 60% in *StNRL1*-56 and *StNRL1*-22 mutant lines, respectively (**Figure 4**).

### *StNRL1* mutants showed enhanced late blight resistance

**Figure 5:**
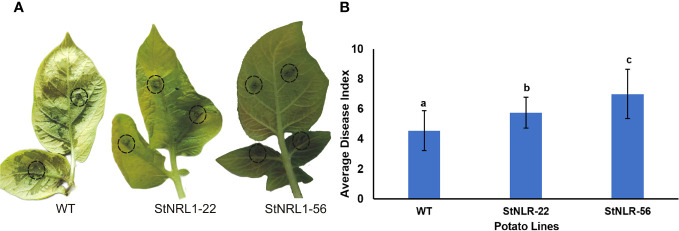
Light blight disease assay of detached leaves challenged with *P. infestans*. The detached leaves of wild-type plants, the tetra-allelic mutant plants (*StNRL1*-56), and the heterozygous *StNRL1* mutant lines were inoculated with 10 μl of zoospore suspension on both sides of leaves abaxial for 10 days. **(A)** Representative leaves showing increased lesion size. **(B)** The average disease index score was calculated from four repetitions of three leaflets. Bars represent means ± SD. The average disease index scores were compared by one-way ANOVA followed by Fisher’s LSD, lowercase letters differ significantly at P < 0.05.

To assess the significance of *StNRL1* gene knockout in T1 potato CRISPR lines, detached leaf assays were performed. The tetra-allelic *StNRL1*-56 line and *StNRL1*-22 heterozygote lines with a high level of *StNRL1* expression reduction were compared to the wild-type Agria plants as control (**Figure 5A**). The infection lesion diameter was determined at 7 days post inoculation (dpi) from leaves challenged with *P. infestans*. Small and large lesions were observed on the inoculated leaves of the control and *StNRL1*-56 knockout line, respectively. The lesion size on the heterozygote mutant line was more similar to that of the control plant. At 7 dpi, the inoculated parts of control plant leaves were blighted showing visible sporulation, whereas on the *StNRL1*-56 mutant line leaves, the sporulation was not visible by naked eye, suggesting that in the knockout *StNRL1*-56 mutant line, susceptibility to *P. infestans* was reduced as evidenced by the sizes of the lesions (**Figure 5B**). These results were in agreement with the transient silencing of *NbNRL1* in *Nicotiana benthamiana* (Yang et al., 2016) where silencing of *NbNRL1* accelerated INF1 cell death and modulated *P. infestans* leaf colonization (Yang et al., 2016).

The *StNRL1* protein belongs to the nonphototrophic hypocotyl3/root phototropism2-like (NPH3/RPT2) protein family in *Arabidopsis*. Overexpression of *NRL1* gene led to the suppression of INF1-mediated cell death and increased leaf colonization by *P. infestans*, indicating that *NRL1* acts as a susceptible factor and promotes the late blight disease. All members of this family contain N-terminal BTB/POZ (19–122), a central NPH3 domain, and a C-terminal coiled-coil domain. The BTB domain is required for dimerization of proteins and selection of substrates for ubiquitination. Thus, as evidenced by sequencing in our CRISPR lines, mutations in conserved pocket residues in the BTB/POZ domain prevented *NRL1*-mediated dimerization *in planta*. The conserved residues (Asp28 and Lys42) in *StNRL1* are required for *StNRL1* to interact with *StSWAP70*, as Asp28 (D28) to Asn (N) and Lys42 (K42) to Gln (Q) substitutions in *StNRL1* hinder *StNRL1* and *StSWAP70* interaction (He et al., 2018). In this study, the large deletion already omits the pivotal amino acids in the CRISPR mutant lines; consequently, *StNRL1* dimerization is impaired and *P. infestans* is no longer able to colonize plant tissues.

### *StNRL1* knockout promoted susceptibility to early blight

**Figure 6:**
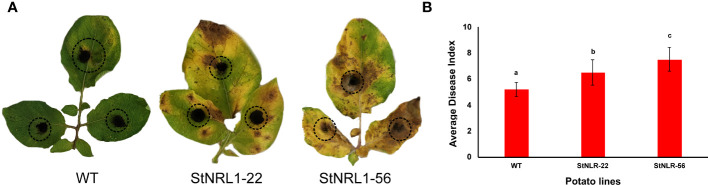
Early blight disease assay of detached leaves. A 1-cm^2^ agar block of the *A*. *alternata* culture was placed on both sides of wild-type plants and homozygous *StNRL1*-56 and heterozygous *StNRL1* mutant lines for 10 days. **(A)** Extended chlorosis in the leaves of CRISPR lines is highly visible. **(B)** The average disease index score was calculated from three repetitions of three leaflets. Bars represent means ± SD. The average disease index scores were compared by one-way ANOVA followed by Fisher’s LSD, lowercase letters differ significantly at P < 0.05.

Since early blight is a much larger threat to potato cultivation in Iran, to this end, the *StNRL1*-56 and *StNRL1*-22 CRISPR mutant lines were also challenged with *A. alternata*, the causal agent of the early blight disease (**Figure 6A**). Surprisingly, the detached leaf assay analyses showed that the T1 CRISPR mutant plants were more susceptible to early blight than control plants as can be seen from the size of lesions (**Figure 6B**). It can be inferred from these results that, presumably, *NLR1* protein plays a dual function. This may imply that *A. alternata* effectors perturb resistance signaling pathways triggered by or dependent on *NLR1* expression. Toxin effectors secreted by necrotrophic fungi like *A. alternata* can target a host’s central signal regulator to trigger R-gene-mediated resistance, thereby increasing host susceptibility (Ghozlan et al., 2020). In contrast to biotrophs, R-gene-mediated response to some host-specific necrotrophs (HSNs) may lead to host susceptibility (Ghozlan et al., 2020). A gene-for-gene relationship between host-specific toxins (HSTs) and the host R-proteins may play a role as the effector-triggered susceptibility complex (Ghozlan et al., 2020). For example, *Pyrenophora tritici-repentis* as a necrotrophic fungus secretes PtrToxA and subverts the host resistance mechanisms. PtrToxA activates host responses by interaction with the R-proteins of *Tsn1* gene, a classical plant R-gene that is associated with resistance responses to biotrophic pathogens upon wheat tan spot disease (Adhikari et al., 2009). *Cochliobolus victoriae*, the causal agent of Victoria blight, is another necrotrophic pathogen that exclusively appeared on oat plants carrying the R-gene Pc-2, which confer disease resistance against the biotrophic fungus *Puccinia coronata* (Stotz et al., 2014). Such results confirm previous studies showing that the type of resistance differs between biotrophic and necrotrophic pathogens (Liao et al., 2022). This is because biotrophic pathogens obtain nutrients and energy from living cells and tissues and often secrete limited amounts of cell wall-degrading enzymes and effectors to suppress the host immune system, whereas necrotrophic pathogens obtain their energy from dead or dying cells, which they kill before or during colonization (Wang et al., 2014; Rajarammohan, 2021). Hemibiotrophic pathogens initially invade living cells before switching to a necrotrophic lifestyle to obtain nutrients from killing the host cells (Rajarammohan, 2021).

The plant innate immune system includes PAMP-triggered immunity (PTI) and effector-triggered immunity (ETI) (Wang et al., 2014). PTI is an innate immunity triggered in plants when PAMPs are recognized by pattern recognition receptors (PRRs) (Wang et al., 2014). Although PTI is the predominant immunity system in biotrophs and hemibiotrophs, it plays only minor roles in resistance to necrotrophs (Liao et al., 2022). In contrast, ETI is triggered by recognition of pathogen effectors by host proteins encoding the disease resistance proteins (R proteins). ETI often leads to a rapid and robust response that is often referred to as a hypersensitive reaction (HR) showing localized host cell death (Jones and Dangl, 2006; Wang et al., 2014). As a result, biotrophic pathogens cannot survive and infect plant tissues (Wang et al., 2014), whereas necrotrophic fungi secrete HSTs to activate ETI, eventually leading to HR cell death (Wang et al., 2014).

In conclusion, plant breeders aim to improve disease-resistant crops by either pyramiding the R-genes or knocking out the S-genes. With development of advanced genome editing approaches, the knocking out of S-genes is a promising method to decrease the susceptibility of crop cultivars to plant diseases. We used a CRISPR/Cas9 system as a powerful tool to knock out the S-gene *StNRL1* in potatoes. We showed that *StNRL1* gene is involved in *P. infestans* infection and resistant to *A. alternata*. Our study confirmed that the defense mechanisms can be different between biotrophic and necrotrophic organisms and the CRISPR/Cas9 system is a powerful tool to study and characterize S-genes. A more refined and sophisticated genome editing strategy based on new genome editing approaches will be required to extend the spectrum of resistance to most plant fungal and bacterial diseases. At the same time, to generate disease-resistant potato cultivars, improved transformation-regeneration protocols will be required to maximize transformation efficacy as well as *in vitro* potato plant regeneration. Nonetheless, it would be vital to establish transgene-free potato elite cultivars because it is still challenging to recover a Cas9-free mutated line blaming the tetraploid nature of potato.

## Data availability statement

The original contributions presented in the study are included in the article/**Supplementary Material**. Further inquiries can be directed to the corresponding author.

## Author contributions

FN: Supervision, Writing – review & editing, Software. MN: Data curation, Formal analysis, Investigation, Methodology, Project administration, Software, Validation, Writing – original draft. AI: Supervision, Writing – review & editing. RA: Funding acquisition, Methodology, Resources, Supervision, Validation, Investigation, Writing – review & editing. HP: Writing – review & editing.
