# Corrigendum: CRISPR/Cas *StNRL1* gene knockout increases resistance to late blight and susceptibility to early blight in potato

**DOI:** 10.3389/fpls.2024.1435731

**Published:** 2024-06-07

**Authors:** Moshen Norouzi, Farhad Nazarain-Firouzabadi, Ahmad Ismaili, Rahim Ahmadvand, Helen Poormazaheri

**Affiliations:** ^1^ Production Engineering and Plant Genetics Department, Faculty of Agriculture, Lorestan University, Khorramabad, Iran; ^2^ Associate Professor, Seed and Plant Improvement Institute, Agricultural Research, Education and Extension Organization, Karaj, Iran; ^3^ Department of Biology, Shahr-e-Qods Branch, Islamic Azad University, Tehran, Iran

**Keywords:** *Alternaria alternata*, CRISPR/Cas9, effector Pi02860, NPH3/RPT2-LIKE1 protein, *Phytophthora infestans*, susceptible gene

**Incorrect Author Name**

In the published article, the author’s name was incorrectly written as Mohsen Nourozi. The correct spelling is Moshen Norouzi.

**Incorrect Funding**

In the published article, there was an error in the Funding statement. The statement read as: “The author(s) declare that no financial support was received for the research, authorship, and/or publication of this article.” The correct Funding statement should read as follows:

“The authors declare that financial support was received for the research, authorship, and/or publication of this article. This work was partly supported by the Iran National Science Foundation (INSF) [Grant Number 96012156.”

The authors apologize for this error and state that this does not change the scientific conclusions of the article in any way. The original article has been updated.

