# Supplementary material for: CRISPR/Cas StNRL1 gene knockout increases resistance to late blight and susceptibility to early blight in potato
Source: Front Plant Sci. 2024 Jan 18;14:1278127. doi: 10.3389/fpls.2023.1278127 (PMC10830690; doi:10.3389/fpls.2023.1278127)
Supplement: Supplementary file 1 [file DataSheet_1.docx]

Supplementary Figures


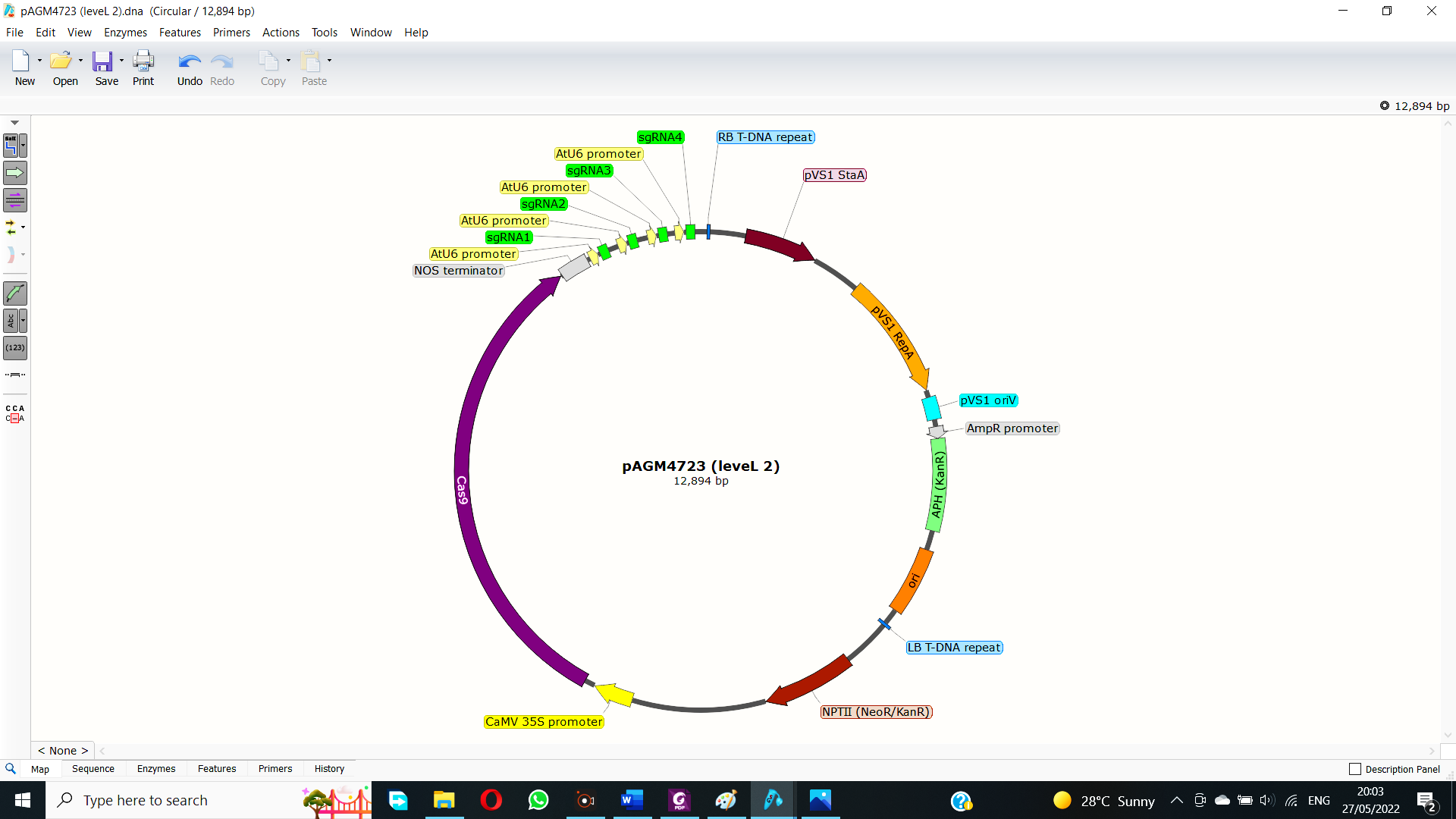


Supplementary figure 2. Schematic representation of a CRISPR.Cas9 construct used for potato transformation to target StNRL1 gene.


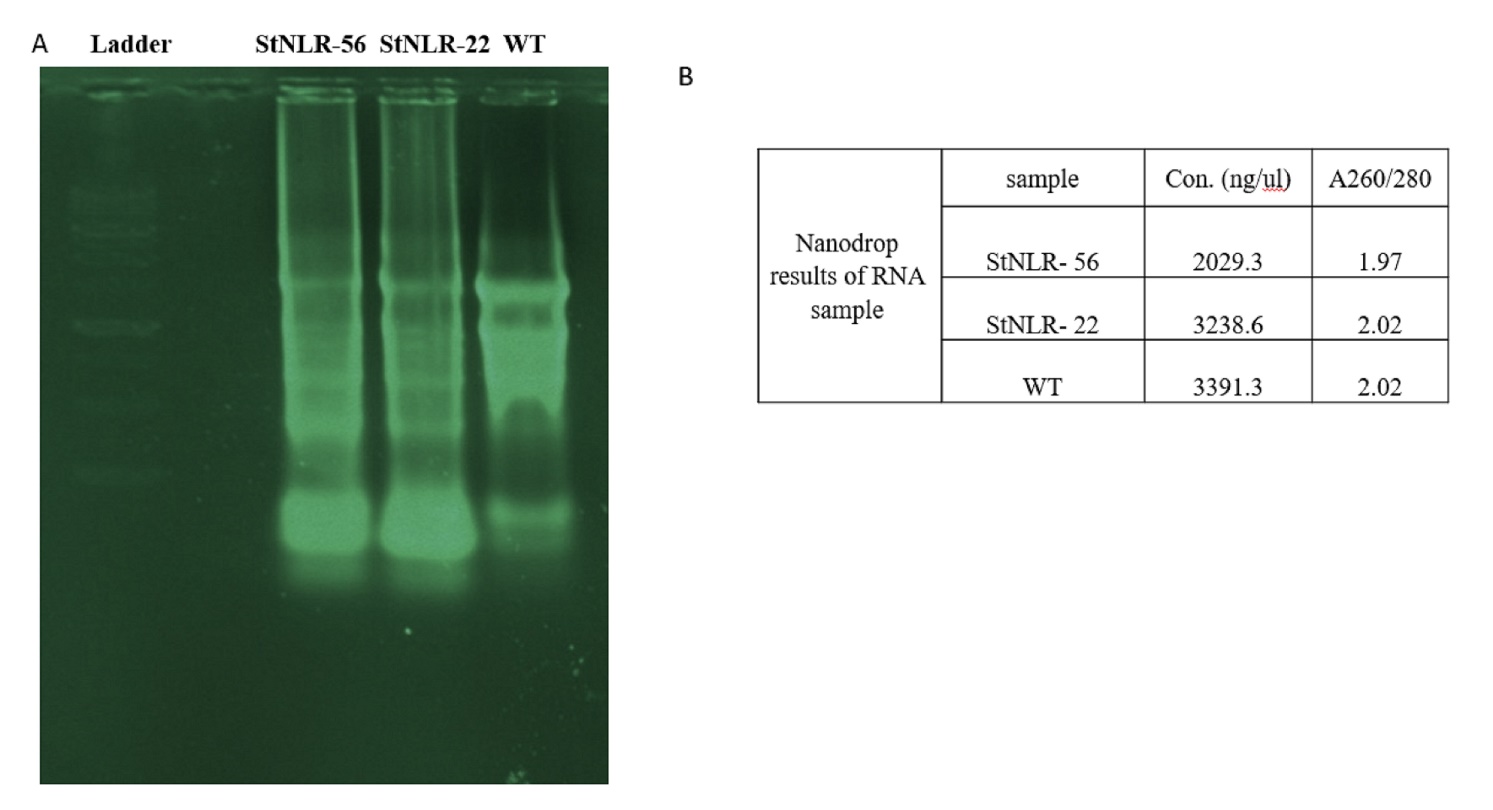


**Supplementary figure 2.** The quality and quantity of extracted total RNA were evaluated using agarose gel electrophoresis and Nanodrop spectrophotometer analyses.
